# Supplementary material for: TRRAP Enhances Cancer Stem Cell Characteristics by Regulating NANOG Protein Stability in Colon Cancer Cells
Source: Int J Mol Sci. 2023 Mar 26;24(7):6260. doi: 10.3390/ijms24076260 (PMC10094283; doi:10.3390/ijms24076260)
Supplement: Supplementary file 1 [file ijms-24-06260-s001.zip › ijms-2236092-supplementary.pdf]

Supplemental Information

TRRAP Enhances Cancer Stem Cell Characteristics by Regulating NANOG Protein Stability in Colon Cancer Cells

Kyung-Taek Kang[1], Min-Joo Shin[1], Hye-Ji Moon[1], Kyung-Un Choi[2], Dong-Soo Suh[3] and Jae-Ho Kim[1,4*]

[1]Department of Physiology, College of Medicine, Pusan National University, Yangsan 50612, Gyeongsangnam-do, Republic of Korea;

[2]Department of Pathology, College of Medicine, Pusan National University, Yangsan 50612, Gyeongsangnam-do, Republic of Korea;

[3]Department of Obstetrics and Gynecology, School of Medicine, Pusan National University, Yangsan 50612, Gyeongsangnam-do, Republic of Korea;

[4]Convergence Stem Cell Research Center, Pusan National University, Yangsan 50612, Gyeongsangnam-do, Republic of Korea
*Correspondence: jhkimst@pusan.ac.kr; Tel.: +82-51-510-8073; Fax: +82-51-510-8076

Supplemental Materials and Methods

Coomassie blue staining

After electrophoresis, gel was washed 3 times, 5 minutes each in deionized water. Gel was stained with Coomassie Blue R-250 Staining Solution (Enzynomics, Daejeon, Republic of Korea) for 1 hour in room temperature. Then gel was distained for 2 hours with 50% methanol, 10% acetic acid, 40% deionized water in room temperature.

Elution of TAP-NANOG and protein interactions

After pulldown of TAP-NANOG with streptavidin beads, beads were washed 3 times for 5 minutes. TAP-NANOG was eluted for 30 minutes at 4°C with streptavidin elution buffer (Agilent Technologies, Santa Clara, California, USA). Eluted TAP-NANOG was incubated with FLAG-control or FLAG-TRRAP for 2 hours followed by a pull-downed with anti-FLAG M2 affinity beads. Beads were washed 3 times and boiled for 10 minutes with sample buffer.

Supplementary Figures

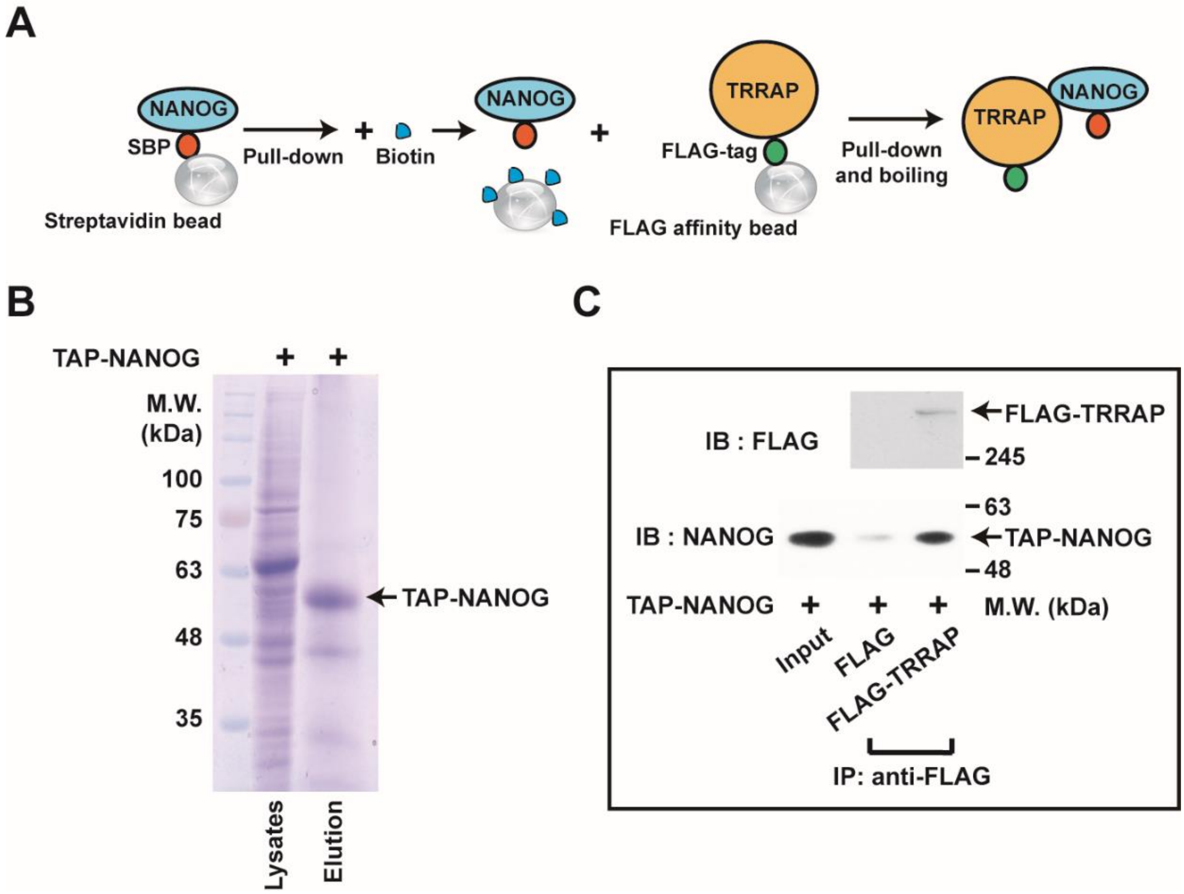

Figure S1. Direct interaction of NANOG with TRRAP. **(A)** Schematic illustration for purification of TAP-NANOG and pull-down experiment using FLAG-TRRAP immunoprecipitants. TAP-NANOG was overexpressed in HEK293 cells, purified with Streptavidin affinity purification, and the TAP-NANOG protein was eluted from the Streptavidin agarose bead by treatment with elution buffer containing biotin. The purified TAP-NANOG protein was incubated with the FLAG-TRRAP immunoprecipitants, followed by pull-down of the FLAG-TRRAP-associated TAP-NANOG protein complex by spin down experiment. SBP: Streptavidin binding peptide. **(B)** The cell lysates of TAP-NANOG-overexpressed HEK293 cells and the purified TAP-NANOG protein were separated by SDS-PAGE, followed by staining with Coomassie Blue R-250. The arrow indicates the protein band of TAP-NANOG. **(C)** Direct binding of the purified NANOG with FLAG-TRRAP. The cell lysates of TAP-NANOG-overexpressed HEK293 cells (input) were incubated with the immunoprecipitants of FLAG or FLAG-TRRAP, followed by pull-down experiment for isolation of the TRRAP-bound NANOG. The input and immunoprecipitants were analyzed by western blotting analysis of the FLAG-TRRAP and TAP-NANOG.
